# DIFFERENCES IN SELF-REGULATION BETWEEN OLDER DRIVERS WITH AND WITHOUT DEMENTIA

**DOI:** 10.1093/geroni/igz038.1240

**Published:** 2019-11-08

**Authors:** Jennifer S Zakrajsek, Lisa J Molnar, David W Eby

**Affiliations:** 1 University of Michigan Transportation Research Institute, Ann Arbor, Michigan, United States; 2 University of Michigan Transportation Research Institute and Center for Advancing Transportation Leadership and Safety, Ann Arbor, Michigan, United States

## Abstract

Given the high current prevalence of dementia and expected increases in the coming years, it is increasingly important to understand and address the process of transitioning from driving to non-driving among older drivers with dementia and the potential role of self-regulation (e.g., avoiding challenging driving situations). The purpose of this Alzheimer’s Association-funded study was to examine differences in self-regulation between older drivers with and without dementia. Fifty-two active drivers age 65 or older (16 with dementia; 36 healthy controls), completed a validated self-regulation questionnaire and standardized assessment of cognition, vision, and psychomotor skills. The dementia group reported some awareness of cognitive challenges (lower driving-related abilities to remember things and concentrate on multiple things at a time than the comparison group). Similarly, the dementia group performed significantly worse on memory tasks and complex reaction time in the objective assessment. Both groups were confident they could drive safely where they needed to go. However, the dementia group reported being less comfortable driving in several situations (unprotected left turns, unfamiliar areas, alone, rush hour traffic, backing up). Despite lower driving comfort among the dementia group, there were few differences in self-regulation between the groups. The only statistically significant difference was that more of the dementia group had reduced their driving in the past year. There were no differences in self-regulation between groups for any of the specific driving situations for which the dementia group reported feeling less comfortable. Full study results will be presented along with implications for programs and future research.

